# Insights gained into respiratory infection pathogenesis using lung tissue metabolomics

**DOI:** 10.1371/journal.ppat.1008662

**Published:** 2020-07-14

**Authors:** Jean A. Bernatchez, Laura-Isobel McCall

**Affiliations:** 1 Skaggs School of Pharmacy and Pharmaceutical Sciences, University of California, San Diego, La Jolla, California, United States of America; 2 Center for Discovery and Innovation in Parasitic Diseases, University of California, San Diego, La Jolla, California, United States of America; 3 Department of Chemistry and Biochemistry, University of Oklahoma, Norman, Oklahoma, United States of America; 4 Department of Microbiology and Plant Biology, University of Oklahoma, Norman, Oklahoma, United States of America; 5 Stephenson Cancer Center, University of Oklahoma, Oklahoma City, Oklahoma, United States of America; 6 Laboratories of Molecular Anthropology and Microbiome Research, University of Oklahoma, Norman, Oklahoma, United States of America; University of Kentucky, UNITED STATES

Respiratory infections have long represented a serious threat to humanity, given their relative ease of dissemination via aerosols. The current pandemic caused by the severe acute respiratory syndrome coronavirus 2 (SARS-CoV-2) and recent outbreaks of related coronaviruses and influenza continue to highlight the importance of studying the pathogenesis of these diseases to better prepare ourselves for the next threat. Elucidating the metabolic determinants of severe versus mild disease states in the lung during respiratory infections may hold the key to the development of therapeutics to modulate symptom and disease severity. Metabolomics and the related field of lipidomics seek to analyze the global changes of small molecule effectors of gene expression and lipids in living systems, respectively. Two principal techniques are used to study these changes: mass spectrometry (MS) and nuclear magnetic resonance (NMR) spectroscopy. The first technique utilizes the mass-to-charge ratio of molecular ions to help elucidate molecular structure, whereas the second technique utilizes the differences in local magnetic fields about the nuclei of atoms in a molecule to help determine the chemical structure [1]. In this Pearl, we review the advances in the fields of untargeted metabolomics and lipidomics as they pertain to the study of lung tissue, in respiratory infectious diseases. For in vitro or ex vivo immunometabolomic studies of respiratory pathogens or for targeted metabolomic analyses, we refer the reader to excellent reviews on these topics (e.g., Goodwin and colleagues [2], du Preez and colleagues [3], Rao and colleagues [4]).

## Viral infections

Studies of the lung metabolome in viral infections have focused predominantly on influenza virus (6 studies) [5, 6, 7, 8, 9, 10], with one additional study on respiratory syncytial virus (RSV) [11]. Influenza virus studies were performed in ferrets (1 study) [5] and C57BL/6 mice (5 studies) [6, 7, 8, 9, 10], with all but one study [8] using MS. These differences in animal model, viral strain, instrumentation, and data acquisition and analysis parameters, as well as the use of different infection time points, likely account for the limited overlap between these studies. Indeed, only uridine, sphingosine, sphinganine, and kynurenine were found increased in more than one study (Cui and colleagues [6] and Chandler and colleagues [9]), and adenosine monophosphate (AMP) and threonine showed opposite trends depending on the study [5, 6, 9]. However, common trends include alterations in amino acids and related molecules [5, 6, 9], in some nucleosides, nucleotides, and analogs [5, 6, 9], and in select lipids [5, 6, 9, 10], increases in carbohydrates and related molecules (Tisoncik-Go and colleagues [5] and Cui and colleagues [6]) and decreases in mannitol, myo-inositol, and glyceric acid [5] (Table 1). Changes in amino acids, lipids, and nucleosides/nucleotides likely reflect the consequences of viral manipulation of host metabolism to favor the production of new viral particles, whereas modulation of pro-inflammatory (e.g., sphingosine, which is metabolized to sphingosine-1-phosphate) and anti-inflammatory metabolites (e.g., kynurenine) contribute more indirectly to disease pathogenesis.

**Table 1:** Lung metabolomics studies.

Reference	[5]	[6]	[7]^1^	[8]^1^	[9]	[10]	[11]	[12]	[13]	[14]	[15]	[16]	[17]	[18]	[19]	[20]
**Pathogen**	influenza virus	influenza virus	influenza virus	influenza virus	influenza virus	influenza virus	RSV	*M*. *tuberculosis*	*M*. *tuberculosis*	*M*. *tuberculosis*	*M*. *tuberculosis*	*P*. *aeruginosa*	*P*. *aeruginosa*	*F*. *tularensis*	*C*. *neoformans*	*A*. *fumigatus*
**Pathogen strain**	Influenza A: 1918, CA04 (H1N1)	Influenza A: PR8 (H1N1)	Influenza A: CA04 (H1N1)	Influenza A: PR8 (H1N1)	Influenza A: CA04 (H1N1)	Influenza A: PR8, CA04 (H1N1), Vietnam/1203/04 (H5N1)	A2	H37Rv	4619, 4233, 4147, 3393	H37Rv	H37Rv	CHA, CHAΔpopBD	CHA	live vaccine strain	VPB571–015	1059 CCF
**Model**	ferrets	mice	mice	mice	mice	mice	mice	guinea pig	guinea pig	mice	mice	mice	mice	mice	rats	rats
**Model strain**	-	C57BL/6	C57BL/6	C57BL/6	C57BL/6	C57BL/6	BALB/c	Hartley	Hartley	C57BL/6	C57BL/6	C57BL/6	C57BL/6	ICR	Fisher 344	Lewis
**Method**	LC-MS; GC-MS	LC-MS	LC-MS	NMR	LC-MS	LC-MS	LC-MS	NMR	NMR	NMR	CE-MS, LC-MS, GC-MS	NMR	TOF-SIMS	DESI-MS	NMR	LC-MS, MALDI-MSI
**Timepoint (days)**	1, 3, 8	0, 6, 10, 14, 21, 28	4, 8	9	10	0.25, 0.5, 1, 2	1, 2, 4, 6, 7, 8, 10	15, 30, 60	30, 60	28	28, 63	0.6 to 0.75	0.7	6	5–7	3
**Increased**	**Amines**															
	-	Sphinganine, sphingosine	-	-	Sphinganine, sphingosine	-	Putrescine	-	-	-	Putrescine, N-acetylspermidine, spermidine, spermine	-	-	-	-	-
	**Amino acids, peptides, analogues, and derivatives**															
	-	Leucine, methionine, phenylalanine, L-2-aminoadipic acid, taurine, pantothenic acid, kynurenine, hydroxyindole acetic acid, N-acetyl-l-aspartic acid, leucyl-aspartate, leucyl-glycine, glycyl-tyrosine, leucyl-proline, glycyl-phenylalanine, S-lactoylglutathione, pyruvic acid	-	-	Serine, threonine, tryptophan, kynurenine, kynurenic acid, formylkynurenine, indole, indole acetaldehyde, hydroxyindoleacetic acid, methylene oxindole, glutamyl-lysine	-	Arginine, citrulline, histidine, kynurenine, indole, ornithine	Alanine, aspartate, glutamate, reduced glutathione, oxidized glutathione, creatine, TMAO^2^, betaine	Alanine, aspartate, creatine, betaine/trimethylamine N-oxide, glutathione	Alanine, aspartate, glutamate, glutamine, isoleucine, leucine, lysine, phenylalanine, tyrosine, creatine, betaine, taurine, glutathione	Arginine, aspartate, citrulline, cysteine, glutamine, glycine, ornithine, kynurenine, methylhistidine, oxidized glutathione, reduced glutathione, hypusine, S-adenosylmethionine, S-lactoylglutathione, trans-4-hydroxyproline, TMAO, deoxyhypusine, ergothioneine, galactosylhydroxylysine	leucine or isoleucine, valine	-	-	-	-
	**Carbohydrates and carbohydrate conjugates**															
	Fructose	D-mannose-1P, N-acetylneuraminic acid	-	-	-	-	-	Dihydroxyacetone	-	-	Glucose-6-phosphate, N-glycolylneuraminate	Glucose	-	-	Trehalose, mannitol	-
	**Lipids and lipid-like molecules**															
	Palmitic acid, glycerol-3-phosphate, PC(38:4), PC(38:5), PC(38:6), PE(36:2), PE(38:4), PE(38:5), PE(40:4), PE(40:6), TG(18:1/18:1/22:4)	PC(32:1), PC(38:1), PC(44:6), PE(18:0), PE(22:4), MG(22:6), acetylcarnitine, hydroxybutyrylcarnitine, propionylcarnitine, butenylcarnitine, hydroxyhexanoylcarnitine, octenoylcarnitine	-	-	PCs, PS, phosphatidylglycerols, PEs	PGE2, LXA4 (strain-dependent), PGD2 (strain-dependent), 15-deoxy-PGJ2, PGF2a, 6-keto-PGF1a, 5-HEPE (strain-dependent), 8-HEPE (strain-dependent), 12-HEPE, PGE3	-	PC, GPC	Phosphocholine, GPC	PC, PE	Linoleic acid, palmitoleic acid, PE, acetylcanitine propionylcanritine, butyrylcarnitine, hexanoylcarnitine	-	Linoleic acid, cholesteryl sulfate	PC(34:1), PC(36:2), PC(36:3), PC(36:4), PI(38:4)	GPC	-
	**Nucleosides, nucleotides, and analogues**															
	-	Guanosine, xanthine, thymidine, uridine, succinyladenosine, AMP, GMP, TMP, cAMP, dAMP, dCMP, ADP, UDP-N-acetylglycosamine, GDP-l-fucose	-	-	Cytidine, cytosine, inosine, hypoxanthine, uridine	-	-	-	-	AMP, UDP-glucose, uridine, xanthine, uracil	Cytidine, uracil, uridine CMP, GDP, CDP-ethanolamine, 5-methylthioadenosine	-	-	-	-	-
	**Organic acids**															
	Lactic acid	Glutaconic acid	-	-	-	-	-	Lactic acid, acetic acid	Lactic acid, acetic acid	Lactic acid, succinic acid, itaconic acid	Lactic acid, malic acid, itaconic acid	Succinic acid	-	-	Acetic acid	-
	**Other**															
	-	-	-	-	-	-	6-hydroxymelatonin	-	-	Acetaldehyde	Nicotinamide	-	-	-	Ethanol	*Aspergillus* siderophore ferricrocin (FC), *Aspergillus* siderophore desferri-FC
**Decreased**	**Amines**															
	Ethanolamine	-	-	-	-	-	-	-	-	-	-	-	-	-	-	-
	**Amino acids, peptides, analogues, and derivatives**															
	Threonine valine, aminomalonic acid, hypotaurine	-	-	-	Asparagine, trimethylamine oxide, methylniacinamide, pyrroline-5-carboxylate, hexaprenyl hydroxybenzoic acid, hydroxy-l-tryptophan	-	Glycine, desaminotyrosine, xanthurenic acid, serotonin, 5-hydroxyindoleacetic acid, aminoadipic acid, hydroxyphenyllactic acid	-	-	-	Serotonin	Glycine, glutathione	-	-	-	-
	**Carbohydrates and carbohydrate conjugates**															
	Mannitol, myo-inositol, glyceric acid	-	-	-	-	-	-	-	-	Glucose	-	-	-	-	-	-
	**Lipids and lipid-like molecules**															
	PC(16:0), PC(18:1), PE(38:4), PI(36:2), TG(16:1/16:1/18:1), TG(16:0/16:0/18:1), TG(16:0/16:1/18:1), TG(16:1/18:2/18:2), TG(16:0/16:1/18:2)	-	-	-	PE, dodecanol	12-HETE (strain-dependent), 15-HETE (strain-dependent), 17-HDoHE (strain-dependent), PD1 (strain-dependent), 5-HETE (strain-dependent), 5-oxo-ETE, 9-HETE, 19-HETE, 16-HETE, 20-HETE	-	Phosphatidylcholine	-	-	-	GPC	-	PG(34:1)	-	-
	**Nucleosides, nucleotides, and analogues**															
	Uracil	CMP, UDP	-	-	Adenine, AMP, allantoin	-	-	-	-	NAD+, NADP+	Adenosine, UMP	-	-	-	-	-
	**Organic acids**															
	Urea	-	-	-	-	-	-	-	-	Oxaloacetic acid, fumaric acid	Pyruvic acid	-	-	-	-	-
	**Other**															
	-	-	-	-	Amino propanol, trimethylaminoacetone, bis-(2-hydroxypropyl)amine	-	-	Choline	-	-	-	Ascorbate	-	-	-	-

^1^Studies only investigated the impact of obesity on the metabolome.

**Abbreviations:** ADP, adenosine diphosphate; AMP, adenosine monophosphate (cAMP, cyclic AMP; dAMP, deoxyAMP); CE-MS, capillary electrophoresis–mass spectrometry; CMP, cytidine monophosphate (dCMP, deoxyCMP); DESI-MS, desorption electrospray ionization-mass spectrometry; GC-MS, gas chromatography-mass spectrometry; GDP, guanosine diphosphate; GMP, guanosine monosphosphate; GPC, glycerophosphocholine; HDoHE, Hydroxydocosahexaenoic acid; HEPE, hydroxyeicosapentaenoic acid; HETE, Hydroxyeicosatetraenoate; LC-MS, liquid chromatography-mass spectrometry; LX, lipoxin; MALDI-MSI, matrix-assisted laser desorption/ionization-mass spectrometry imaging; MG, monoglyceride; NAD, Nicotinamide adenine dinucleotide; NMR, nuclear magnetic resonance; PC, phosphocholines; PE, phosphoethanolamines; PG, prostaglandin; PS, phosphatidylserines; TG, triglyceride; TMAO, Trimethylamine N-oxide; TMP, thymidine monophosphate; TOF-SIMS, time-of-flight-secondary ion mass spectrometry; UDP, uridine diphosphate.

## Bacterial infections

Lung metabolome characterization in bacterial infections has been performed in *Mycobacterium tuberculosis* infection (four studies, two in guinea pigs [12, 13] and two in C57BL/6 mice [14, 15]), using NMR [12, 13, 14] and capillary electrophoresis–mass spectrometry (CE-MS), gas chromatography-mass spectrometry (GC-MS), and liquid chromatography-mass spectrometry (LC-MS) [15]; in *Pseudomonas aeruginosa* infection (two studies in C57BL/6 mice, one using NMR [16] and one using time-of-flight-secondary ion mass spectrometry (TOF-SIMS)MS [17]); and in *Francisella tularensis* infection in ICR mice using desorption electrospray ionization-mass spectrometry (DESI-MS) [18]. Given the common methods employed in most of the *M*. *tuberculosis* studies, significant overlap in infection-induced metabolic shifts was observed, including increases in lactate, glutamate, aspartate, glutathione, and betaine (Table 1) [12, 13, 14, 15]. Increases in oxidized glutathione are likely due to inflammatory processes [12, 15], and increases in amino acids may reflect increased proteolysis [15]. Succinate was also increased in *P*. *aeruginosa* infection, whereas glutathione was decreased [16]. Glucose was also increased by *P*. *aeruginosa* [16] but decreased by *M*. *tuberculosis* infection [14]. Changes in lactate and succinate likely reflect the complex energy requirements associated with pathogen growth (particularly in the context of intracellular infection with *M*. *tuberculosis*) [14, 16]; these metabolites are also immunomodulatory [4, 21].

## Fungal infections

Few studies have investigated small molecule changes in the lung in response to fungal infection. One study detected siderophores produced by *Aspergillus fumigatus* in the lung of infected, immunosuppressed rats [20]. A second study used NMR to show increases in lung and pathogen-derived sugars, lipids, and alcohols in *Cryptococcus neoformans*–infected rats [19]. These likely represent *Cryptococcus* adaptations to survive in the host environment and microbial metabolism [19].

## Implications for drug development

Metabolic modulation has been successfully deployed in several studies as a therapeutic strategy against respiratory infectious diseases, particularly in the context of immunomodulation [4]. For instance, shifting human macrophages to increased states of aerobic glycolysis has been identified as a potential therapeutic target in mitigating *M*. *tuberculosi*s replication in these cells [22]. Also, inhibition of lactate production via targeting of lactate dehydrogenase A rescues RIG-I-like receptor (RLR)-mediated type I interferon (IFN) production and leads to enhanced protection from vesicular stomatitis virus in multiple tissues, including the lung, in mice [23]. Applying existing metabolomic and lipidomic data acquired from studies of respiratory infections to the development of novel metabolism-focus therapies should therefore be strongly considered going forward. This is particularly important for respiratory diseases in which no specific therapies exist, such as SARS, Middle East respiratory syndrome (MERS), and coronavirus disease 19 (COVID-19). Given the severity of these diseases, and the fact that lung metabolic alterations are observed in a broad range of respiratory infections (Table 1), we expect severe coronavirus infections to cause significant changes in the lung metabolome. Indeed, serum metabolomic studies of COVID-19 patients have revealed major alterations in the plasma and serum metabolome and lipidome, with decreased circulating amino acids in particular [24, 25]. Although select amino acids were decreased in lung *P*. *aeruginosa* and influenza virus infection [16] [5], lung amino acids were predominantly increased by respiratory pathogens [6, 9, 11, 12, 13, 14, 15, 16]. In contrast, metabolite shifts common to COVID-19 patient blood and experimental lung infections include increases in some nucleotide metabolites (also observed in the works by Cui and colleagues and Chandler and colleagues [6, 9]) and decreases in allantoin and mannitol (also observed in Tisoncik-Go and colleagues and Chandler and colleagues [5, 9]) [25]. Whether these changes are also observed at the site of COVID-19 tissue damage remains to be determined.

Similarities in in situ metabolic shifts between different pathogens afford the possibility to develop broad-spectrum therapeutic interventions. Such similarities likely represent host tissue adaptations to the metabolic stress caused by pathogen proliferation and the metabolic needs of immune cells, as well as inflammatory signals. Examples of diseases which display similar changes in specific lung metabolites during infection include RSV in BALB/c mice [11] and influenza virus in C57BL/6 mice [9] (indole increased), *M*. *tuberculosis* [12,13] and *Cryptococcus* [19] (acetic acid increased), *P*. *aeruginosa* [16] and *M*. *tuberculosis* [14] (succinate increased), *M*. *tuberculosis* [14], *P*. *aeruginosa* [16] and influenza virus [6] (leucine increased), *M*. *tuberculosis* [14] and influenza virus [5, 6] (lactic acid, taurine, uridine, and phosphatidylethanolamine increased), and RSV [11] and *P*. *aeruginosa* [16] (glycine decreased) (Table 1). Kynurenine was increased by influenza virus [6, 9], RSV [11], and *M*. *tuberculosis* [15] infection, likely due to the fact that it is produced in response to inflammation. Indeed, increased kynurenine is also observed in the serum of severe COVID-19 patients [24]. Broad-spectrum metabolic modulators would be most useful in the context of emerging pathogens in which the rapid development of therapeutic interventions is required. However, this approach is complicated by discrepancies in the metabolomics literature even for a given pathogen. For example, AMP and threonine were each found to be increased in one influenza virus study yet decreased in another [5, 6, 9]. Likewise, comparison between ferret infection with 1918 and CA04 influenza virus strains shows opposite patterns for select phosphoethanolamines and phosphocholines—PE(36:1), PE(36:2), PE(34:2), PC(38:3) [5]. Comparison between mouse infections with influenza virus strains CA04 (H1N1) and Vietnam/1203/04 (H5N1) showed opposite patterns in pro- and anti-inflammatory lipids such as 10S, 17S-dihydroxydocosahexaenoic acid (PD1) [10]. Further follow-up studies must be performed to confirm the robustness of these results and to settle the record for these disease-specific metabolites. Metabolomics and lipidomics are strongly influenced by sample collection (including animal euthanasia methods [26]), sample storage conditions and duration, metabolite extraction, and data acquisition protocols [27]. These are regrettably not always fully described in publications. Likewise, variability in data analysis methods and lack of implementation of standardized metabolite nomenclature makes comparison between studies challenging, although new open tools are helping address this issue [28].

## Conclusions, challenges, and future perspectives

Although there are many metabolomics studies in respiratory infection, the majority have focused on serum, plasma, and bronchoalveolar lavage fluid (BALF), with few studies directly on the affected lung tissue. This is likely driven by the ease of sample access, particularly from human populations; nevertheless, prior studies have detected significant differences in infection-induced metabolic changes between the lung, serum, and BALF. For example, increases in amino sugar and nucleotide sugar metabolism were only observed in the lung during influenza A virus infection [6]. Likewise, lactate was increased in the *M*. *tuberculosis*-infected lung but decreased in the serum [13]. Thus, although biofluids are suitable for biomarker discovery, a complete understanding of the role of metabolism in respiratory infection pathogenesis requires lung tissue analysis. These studies generally involve terminal sample collection only feasible in animal models. However, the results of such animal model studies can be validated in humans using technologies such as positron emission tomography (PET; see Bassetti and colleagues [29] for a review). Clever studies using cystic fibrosis patient sputum samples cultured under in vitro conditions that mimic in vivo lung pH and oxygen gradient shifts are also helping to understand the metabolic shifts associated with human diseases, including differential production of *P*. *aeruginosa* secondary metabolites depending on pH and oxygen levels [30]. Direct studies of the sputum metabolome from cystic fibrosis patients have also revealed higher levels of metabolite diversity and higher peptide levels in patients with more severe disease states from *P*. *aeruginosa*, likely due to higher proteolytic activity in the lung [31].

Lung metabolomics studies have currently been performed only on a restricted list of pathogens (Table 1). It will be necessary to expand these methods to a broader range of diseases and disease models in the future, to identify common versus disease-specific metabolic alterations. In addition, most lung metabolomics studies do not specify which region of the lung was analyzed. Recent lung spatial metabolomics studies have demonstrated spatial variability in bacterial distribution and host and bacterial metabolism between lung regions (e.g., Garg and colleagues [32]). There is therefore a strong need for a spatial component to be added to metabolomic studies of lung infection. A few studies have detected pathogen-derived metabolites such as ergothioneine, trehalose, ferricrocin, and metabolites from bacterial quorum sensing pathways [32, 20, 19]. The remainder of reported metabolites are common to both host and pathogen metabolism; given the relative biomass of host versus microbe, these are likely predominantly reflective of host metabolic processes. Although this focus on host metabolism is understandable in the context of viral infection, a greater understanding of the integration between pathogen and host metabolism depending on the lung regions will be required, facilitated by MS imaging [20] and new spatial metabolomics approaches [32, 33]. Lastly, given the strong connection between metabolism, tissue damage, and immune responses, such studies have great potential to lead to new ways to manage respiratory infections.
